# Dendrochronology reveals different effects among host tree species from feeding by *Lycorma delicatula* (White)

**DOI:** 10.3389/finsc.2023.1137082

**Published:** 2023-09-01

**Authors:** Andrew C. Dechaine, Douglas G. Pfeiffer, Thomas P. Kuhar, Scott M. Salom, Tracy C. Leskey, Kelly C. McIntyre, Brian Walsh, James H. Speer

**Affiliations:** ^1^ Department of Entomology, Virginia Polytechnic Institute and State University, Blacksburg, VA, United States; ^2^ Appalachian Fruit Research Station, United States Department of Agriculture - Agricultural Research Service (USDA—ARS), Kearneysville, WV, United States; ^3^ Pennsylvania State University Extension, Leesport, PA, United States; ^4^ Geography and Geology Department of Earth and Environmental Systems, Indiana State University, Terre Haute, IN, United States

**Keywords:** *Lycorma delicatula*, spotted lanternfly, *Ailanthus altissima*, tree of heaven, dendrochronology, tree core

## Abstract

The spotted lanternfly, *Lycorma delicatula* (White) (Hemiptera: Fulgoridae), was first detected in the United States in Berks County, Pennsylvania, in 2014. Native to China, this phloem-feeding planthopper threatens agricultural, ornamental, nursery, and timber industries in its invaded range through quarantine restrictions on shipments, as well as impacts on plants themselves. The long-term impacts of *L. delicatula* feeding on tree species have not been well studied in North America. Using standard dendrochronological methods on cores taken from trees with differing levels of *L. delicatula* infestation and systemic insecticidal control, we quantified the impact of *L. delicatula* feeding on the annual growth of four tree species in Pennsylvania: *Ailanthus altissima, Juglans nigra, Liriodendron tulipifera*, and *Acer rubrum*. The results suggest that *L. delicatula* feeding is associated with the diminished growth of *A. altissima*, but no change was observed in any other tree species tested. The results also suggest that systemic insecticides mitigate the impact of *L. delicatula* feeding on *A. altissima* growth.

## Introduction

The spotted lanternfly, *Lycorma delicatula* (White) (Hemiptera: Fulgoridae), is native to China and was first detected in the United States, in 2014, in Berks County, Pennsylvania (1). This phytophagous phloem-feeder has over 100 identified host species worldwide and 56 host species confirmed in North America (2). As a phloem feeder, *L. delicatula* has the potential to cause serious economic and ecological impacts (3). In Pennsylvania, *L. delicatula* has proven to be a major pest to grapevines. Some vineyards, with repeated seasons of high pest pressure from *L. delicatula*, have experienced yield losses of up to 90%, and have been subject to triple the number of insecticide applications (4). Studies have shown that some insecticides kill *L. delicatula*, but re-invasion by adult insects from surrounding forests and vegetation into vineyards continues through the late summer and fall (5, 6).

Although much of *L. delicatula* development can occur on cultivated plants, forest and ornamental/shade trees can be obligate hosts for some of the *L. delicatula* life cycle (4). The tree of heaven, *Ailanthus altissima* (Miller) (Sapindales: Simaroubaceae), is an invasive tree species in North America and is a preferred host of *L. delicatula* in its native range. Although *L. delicatula* may not require this tree to complete development, *A. altissima* certainly can constitute a significant proportion of the diet of *L. delicatula* and is a valuable host plant in the insect’s development (7). The *L. delicatula* host range also comprises many economically important North American hardwoods, including black walnut [*Juglans nigra* L. (Fagales: Juglandaceae)], maple [*Acer* spp. L. (Sapindales: Sapindaceae)], oak [*Quercus* spp. L. (Fagales: Fagaceae)], and tulip poplar [*Liriodendron tulipifera* L. (Magnoliales: Magnoliaceae)] (1). The potential economic losses to the forest industry caused by *L. delicatula* have been projected at US$152.6 million per year in Pennsylvania alone (8). These estimates, however, do not fully account for the ramifications of *L. delicatula* invasion on tree health, as many of these effects have not been investigated.

Invasive phloem-feeding insects are a primary cause of disturbance in many forest ecosystems, altering community dynamics, biogeochemical processes, and carbon cycling (9, 10). Phloem sap is composed of carbohydrates and amino acids that are necessary for the production of proteins (11). Depending on phloem nutritional quality, phloem-feeding insects can feed continuously for many hours, ingesting high amounts of phloem sap and excreting excess glucose (11). Large aggregations of *L. delicatula* feeding on a tree effectively remove quantities of important nutrients from the tree manufactured during photosynthesis. In addition, the consumption of phloem sap results in *L. delicatula*’s excretion of honeydew, facilitating sooty mold growth that inhibits plant photosynthesis (12).

Although *L*. *delicatula* feeding can have detrimental effects on tree physiology in some forest species (13), our understanding of, and methodology for, assessing how sap-feeding insects alter tree growth are limited (14). Dendrochronology, the study of dating events using annual tree rings (15), has been used to identify historic defoliation events and beetle outbreaks in forests throughout the United States (16–18). To date, no study has looked into the effect of *L. delicatula* on the radial growth of host trees. In this study, we used dendrochronological methods to quantify the impact of *L. delicatula* feeding on host tree radial growth, and the ability of systemic insecticide treatments to mitigate this impact. The hypothesis we consider is that the presence of *L. delicatula* populations reduces the woody growth of host trees, as reflected by growth rings. This is important for two reasons. First, in the event that *L. delicatula* has a negative effect on the radial growth of economic hosts, there would be an argument for the value of preventative treatment. Second, regarding the effect of *L. delicatula* on the tree of heaven, field observations have revealed visible effects on tree vigor and health; knowledge of these effects could result in the extended lifespan of treated trap trees and our increased understanding of the ecological impacts of this insect on this host.

## Materials and methods

### Study area

To investigate the impact of *L. delicatula* feeding on the radial growth of known host trees, samples were collected from two sites in Pennsylvania where *L. delicatula* has been established in high densities. The Pennsburg site was first documented as containing *L. delicatula* in 2016, and the Blue Marsh Lake site was first documented as containing *L. delicatula* in 2017. The populations of both of these sites increased year over year [personal observations, BW and Brianna Treichler, the United States Army Corps of Engineers (USACE)] following initial infestation and continued to grow throughout 2020 at both sites. “High density” is a relative term and is often relative to the lifecycle stage and corresponding host species. The sites contained clear evidence of *L. delicatula* feeding on common host trees, particularly sooty mold growth on the trunks, cadavers from previous seasons abundant on the ground, and nearby understory stunted or killed by the sooty mold growth to the point of resembling the aftermath of a brush fire. It is not uncommon to document several hundred adult *L. delicatula* per tree in a 2-minute visual count on preferred hosts in the fall. Tree species composition at these sites was primarily mixed deciduous hardwood stands native to the area that have been invaded by the tree of heaven. The typical species at these locations include black walnut (*J. nigra*), red maple (*Acer rubrum* L.), silver maple (*Acer saccharinum* L.), tulip poplar (*L. tulipifera*), black cherry (*Prunus serotine* Ehrh.), sassafras [*Sassafras albidum* (*Nutt.0 Nees*)], mixed oak (red (*Quercus rubra* L.), chestnut (*Quercus montana* Willd.), white (*Quercus alba* L.), and hickories [shagbark—*Carya ovata* (Mill.) Koch; pignut—*Carya glabra* (Mill.) Sweet]. The habitat characteristics where trees were sampled generally consisted of fragmented edge habitats along farm fields or maintained parkland adjacent to roads and trails.

On 7 January 2020, tree cores were collected from Pennsburg, Upper Hanover Township of Montgomery County, Pennsylvania, USA (latitude, longitude: 40.36672, −75.54746). The cores of *A. altissima* (*n* = 10)*, Ac. rubrum* L. (*n* = 8)*, J. nigra* L. (*n* = 8), and *L. tulipifera* (*n* = 5), which had high densities of *L. delicatula* feeding on them, were collected between 2016 and 2019. In Pennsburg, the first trees selected were *A. altissima*, which were divided according to whether they were treated or untreated. Again, larger trees were selected with the expectation that they would provide a longer pre-infestation record. In Blue Marsh Lake, the trees that were selected were *A. altissima*, then treated or untreated (treated trees being previously selected by USACE personnel for treatment based on observed densities and proximity to areas with an increased risk of SLF hitchhiking to new locations on conveyances of park visitors), again with larger trees selected with the expectation of providing a longer pre-infestation record.

On 5 March 2020, *A. altissima* tree cores encompassing three insecticide treatment levels were collected from Blue Marsh Lake Recreation Area in northwest Berks County, Pennsylvania, USA (40.380709, −76.028454), where *L. delicatula* was initially discovered in 2016. The management of *L. delicatula* at Blue Marsh by the Philadelphia District USACE started in 2018 after high densities of adults were observed. The trees were selected for insecticide treatment based on the infestation level of *L. delicatula*. *A. altissima* trees that received 2 consecutive years of insecticide treatment were sprayed on 6 October 2018 and 26 July 2019. The *A. altissima* trees that received a single insecticide treatment were sprayed on 16 August 2019. Afterward, untreated trees still had large numbers of *L. delicatula*. Treated trees were sprayed until runoff with the systemic insecticide dinotefuran (Transtect 70 WSP insecticide; Rainbow Treecare Scientific Advancements, Minnetonka, MN, USA) as a basal bark application at 37.34 g AI/L from the ground to 30–38 cm on the trunk and 360° around the tree. Ten cores were collected from each treatment, for a total of 30 A*. altissima* cores.

### Core collection and laboratory processing

All trees were cored at standard breast height (1.4 m aboveground) using a Jim-Gem® 35-cm increment borer (model 63084; Forestry Suppliers, Jackson, MS, USA) with a core diameter of 5.15 mm, and all trees cored had a diameter at breast height (DBH) longer than 25 cm. The extracted cores were immediately placed in labeled plastic straws lined with hole punches to allow the cores to remain straight while drying. The cores were air-dried on a baking sheet at room temperature for 2 weeks in accordance with standard practice (19).

**Figure 1:**
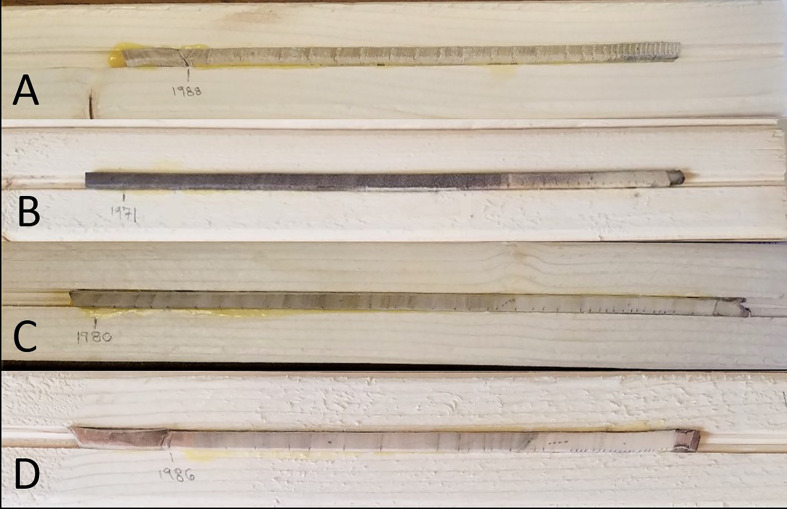
Representative core of each species. *Ailanthus altissima* **(A)**, *Juglans nigra* **(B)**, *Liriodendron tulipifera* **(C)**, and *Acer rubrum* **(D)**, with the year marker representing the first year’s growth.

Once dried, the cores were processed using standard dendrochronological methods (19). The cores were removed from the straws and individually mounted to 25 cm wood blocks with grooves cut down the center to accommodate the core. The groove was approximately 2 mm deep, allowing at least 50% of the core to remain exposed. The exposed surface of each core was then sanded with 220-grit sandpaper using a random orbital sander (DeWalt model DWE6420, Baltimore, MD, USA) for approximately 10–15 seconds to create a flat working surface. Each core was then sanded with progressively finer grit paper (320, 400, and 1,500 grit) for 2 minutes per grit. This was done to remove scratches from the previous grit and create a prepared surface with clearly defined rings and wood cells for dating and measurement under a microscope (20) Representative cores are illustrated in **Figure 1**.

### Core measurement

The tree cores were cross-dated using the list method, a standard process by which narrow rings are matched between cores to ensure accurate dating (21). The ring widths in cores collected from Montgomery County, PA, USA, were measured to the nearest 0.01 mm using a dissecting microscope and Velmex measuring system. A sliding stage was incrementally moved via a small crank and a crosshair in the microscope was used to visually delimit the ring boundaries when taking measurements. The sliding-stage micrometer was connected to a computer and measurements were recorded in MeasureJ2X software (VoorTech Consulting, Holderness, NH, USA).

Due to the university building access restrictions as a result of COVID-19, *A. altissima* cores collected from Berks County, PA, USA, were measured digitally. Cores were placed under a dissecting scope equipped with a nine-megapixel digital camera (SKU: MU900; AmScope, Irvine, CA, USA) that was connected to a computer. Scope calibration and measurements were collected on-screen using AmScope software (version x64, 3.7.7303). The calibration was done at × 1 zoom using a 0.01-mm stage micrometer (SKU: MR096; AmScope). All the ring widths were measured to the nearest 0.01 mm.

After all the ring widths were measured, core dating accuracy was statistically evaluated using the computer program COFECHA (22, 23). COFECHA applies a 32-year cubic smoothing spline across all the data to create a master chronology for each site and species (24). Each tree core series is then compared with the master chronology by splitting it into 50-year segments and using 25 years of overlap to calculate the series intercorrelation for that site and species (24). Potential errors identified by COFECHA were investigated and corrected by re-cross dating. Any cores with unresolvable errors were excluded from further analysis.

### Data standardization: tree size and age

The ring width tends to decrease over time as trees must allocate a greater proportion of resources to wood production to cover an increasing circumference (25). Dendrochronological studies often standardize ring width chronologies to control for varied growth rates among trees of differing sizes and ages (26). To standardize for age–size growth dependencies, raw ring width chronologies were standardized by fitting a negative exponential curve to the data using the computer program ARSTAN (27). ARSTAN was originally developed by Edward R. Cook of Columbia University and has been used since the late 1980s to conduct autoregressive time series standardization of tree ring data (27). If the negative exponential curve did not fit, a horizontal line through the mean was used for standardization (26, 28). The raw ring width value was then divided by the fitted curve value for each measurement, resulting in a dimensionless ring width index (RWI) with an average growth of approximately 1 (25). An RWI > 1 corresponds to greater than average annual growth, whereas a RWI < 1 corresponds to less than average annual growth.

### Data standardization: climatic variables

Standardization has also proven to be useful in understanding the impacts of insects, climate, and other various environmental pressures on tree growth (25). Climatic variables have been shown to influence tree growth (17, 24, 29–31). In this study, we removed the most correlated climate variables from each data set to focus results on the effect of *L. delicatula* feeding. Climate data for both sampling locations were obtained from the National Oceanic and Atmospheric Administration (NOAA) database for Pennsylvania Climate Division 3, Southeastern Piedmont (32, 33). This data set consisted of monthly averages for minimum temperature, maximum temperature, average temperature, precipitation, and Palmer Drought Severity Index (PDSI) values from 1895 to 2019.

To identify the dominant climate variables that altered tree growth, each site and tree species standardized chronology was compared with each climatic parameter using a correlation matrix in Microsoft Excel® (Microsoft Corporation, Redmond, WA, USA) (34). Once the dominant climate variable [the climate variable that most affected tree growth (34)] was identified, all data for that variable were divided by their average to create a dimensionless climate index. To normalize by climate, and thus remove the dominant climate signal, the climate index was subtracted from the standardized chronology (RWI) for each site and species (34).

We attempted to standardize *A. altissima* chronologies obtained from Montgomery County by fitting a negative exponential curve to the raw ring-width data, but later year growth was close to zero and unrealistically skewed the RWIs. Therefore, to equalize the variance across series, we standardized *A. altissima* chronologies in ARSTAN by fitting a horizontal line through the mean, and the distribution of RWIs was then shown to be approximately normal. To maintain consistency all *J. nigra* and *L. tulipifera* series were standardized in ARSTAN by fitting a horizontal line through the mean to equalize variance across the series and the RWI distribution and were shown to be approximately normal (**Table 1**). The same method using ARSTAN was used for all cores.

**Table 1:** Summary of COFECHA results characterizing radial growth of tree species from increment cores.

Site	Species	N_cores_	Mean ring width (mm)	Series intercorrelation*	Mean sensitivity**
Upper Hanover	*Ailanthus altissima*	8	3.80	0.483	0.290
Upper Hanover	*Acer rubrum*	7	1.95	−0.103	0.347
Upper Hanover	*Juglans nigra*	5	2.46	0.307	0.361
Upper Hanover	*Liriodendron tulipifera*	5	5.74	0.592	0.298
Blue Marsh	*Ailanthus altissima*	22	4.79	0.485	0.327

*A measure of how well each tree core series correlates with the master chronology made by COFECHA; a larger number equals a higher correlation.

**A measure of year-to-year variation in tree ring width from 0 to 1. A mean sensitivity of around 0.2 is accepted for climate reconstruction (24).

Growth patterns are characterized among trees of the same species at the same site.

### Data analysis

The RWIs were combined for all cores to form a master chronology for each site and species. Pre- and post-*L. delicatula* infestation years were then compared to determine if there were detectable differences in radial tree growth. Since *L. delicatula* presence was confirmed in the region in 2016, initial populations were likely established in the area in 2015. Thus, tree growth prior to 2015 was considered pre-infestation growth, whereas that from 2015 to 2019 was considered post-infestation growth.

The RWI data were imported into R (The R Foundation for Statistical Computing, Vienna, Austria) (35), where the distribution was checked for normality using the Shapiro–Wilk test. If normality was met, paired t-tests were used to compare the RWI of infested years (2015–2019) to uninfested years (2010–2014) for each site and species. Similarly, a comparison of RWI from earlier uninfested years (2005–2009) to the uninfested years (2010–2014) for each site and species was also created to act as a control and to determine if environmental conditions may have had a significant impact on the mean growth of sampled trees. If normality was not met, RWIs would have been compared using the non-parametric paired Wilcoxon test (36). However, the residuals of all chronologies were shown to be approximately normal, so no Wilcoxon test was needed for analysis.

## Results

### Impact of *Lycorma delicatula* infestation on tree growth

#### Ailanthus altissima

**Figure 2:**
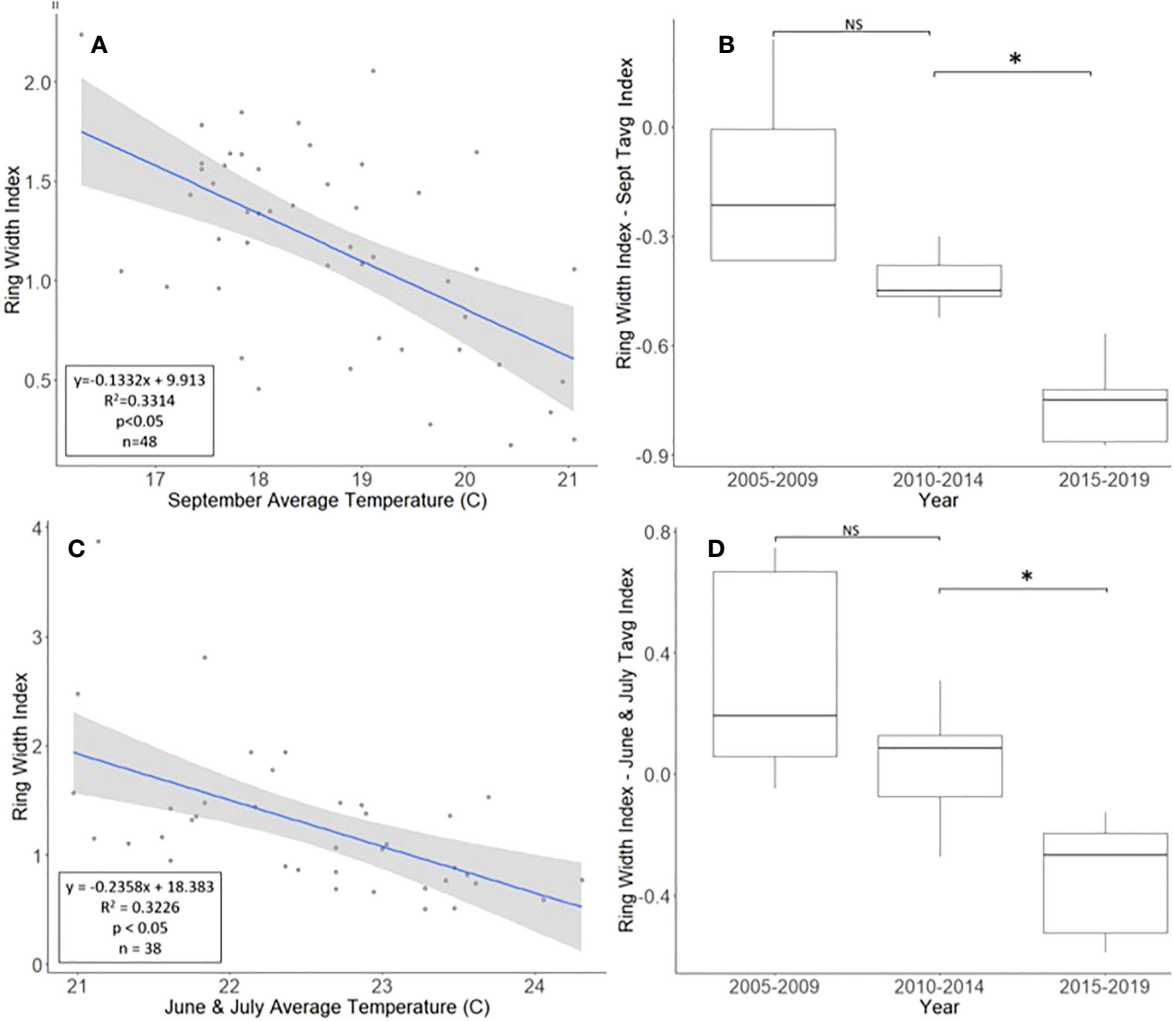
Impact of climate conditions and *Lycorma delicatula* on the ring width index for *Ailanthus altissima* in Upper Hannover Township, PA, USA **(A, B)** and Blue Marsh Recreation Area, Berks County, PA, USA **(C, D)**. Regression analysis of *A altissima* ring width index values and September’s average temperature **(A)**; a comparison of *A altissima* ring width index values with the dominant climate variable removed in years before (i.e., 2005 to 2009 and 2010 to 2014) and after (2015 to 2019) the likely start of *L. delicatula* infestation **(B)**; a regression analysis of *A altissima* ring width index values and the average temperatures for June and July **(C)**; and a comparison of *A altissima* ring width index values with the dominant climate variable removed in trees without insecticide treatment. **(D)**. NS, the difference between means not significantly different from zero; *, the difference between means significantly different from zero (*p* < 0.05).

The *A. altissima* chronologies, obtained from Montgomery County, ranged in length from 16 to 48 years, with a mean length of 30.7 years. For a two-tailed correlation of annual tree ring widths to climate data, with a sample size of 47 years at a confidence level of 0.05, the critical value for Pearson’s correlation coefficient was 0.285 (31). All climatic variables were correlated with the standardized *A. altissima* chronology, and the September average temperature had the highest negative correlation of −0.576. A linear regression analysis was carried out for September’s average temperature as compared with the standardized chronology (**Figure 2A**). The regression analysis showed that approximately 33% [*R* ^2^ = 0.331, degrees of freedom (df) = 47; *p* < 0.001] of the tree’s reduced growth could be attributed to September’s average temperature. After subtracting the normalized climate index from the standardized chronology, a Student’s paired *t*-test showed significantly lower rates of growth from 2015 to 2019 than from 2010 to 2014 (*t* = 4.424, df = 4; *p* = 0.011). The growth from 2005 to 2009 and 2010 to 2014, periods when *A. altissima* was presumed to be uninfested, was not significantly different (*t* = 2.366, df = 4; *p* = 0.077; **Figure 2B**).

#### Juglans nigra

**Figure 3:**
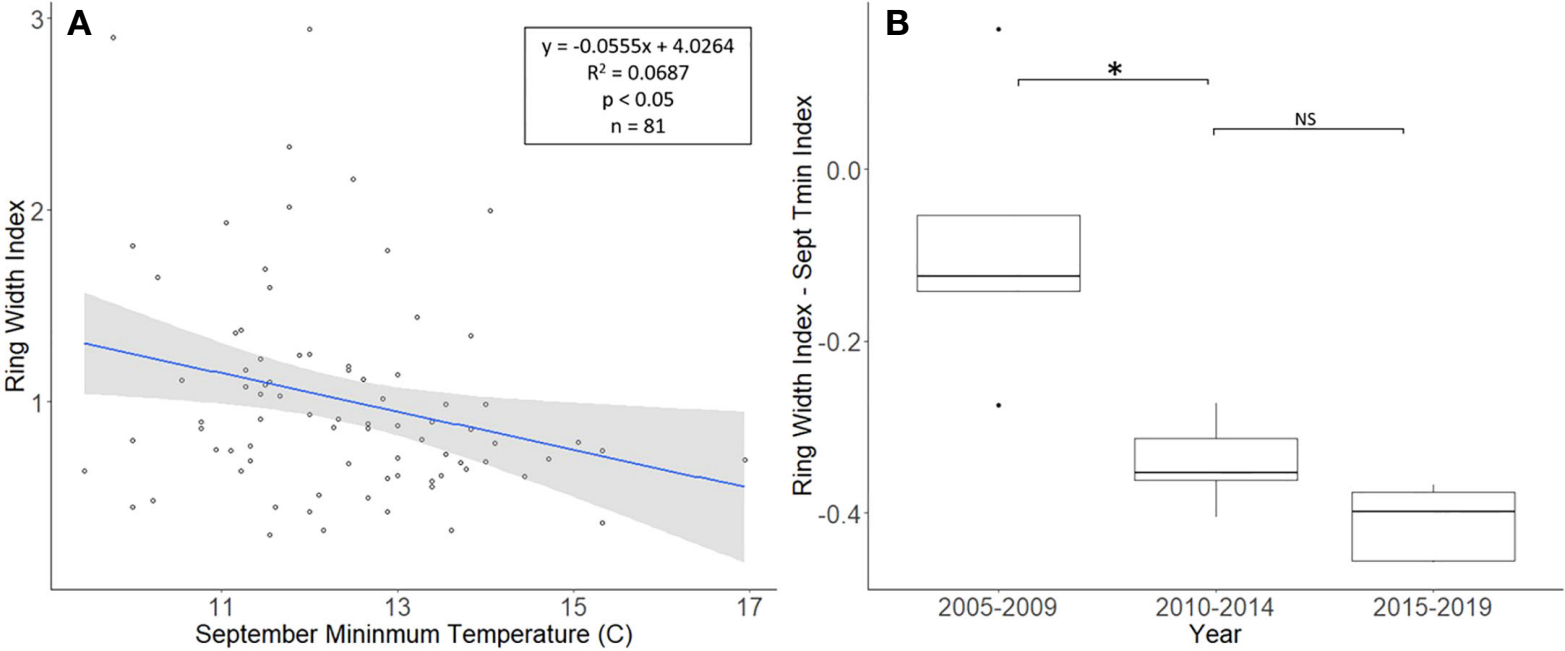
Impact of climate conditions and *Lycorma delicatula* on the ring width index for *Juglans nigra* in Upper Hanover Township, PA, USA. Regression analysis of *J nigra* ring width index values and September’s minimum temperature. **(A)** and a comparison of *J nigra* ring width index values with the dominant climate variable removed in years before (i.e., 2005 to 2009 and 2010 to 2014) and after (2015 to 2019) the likely start of *L. delicatula* infestation. **(B)**. NS, the difference between means not significantly different from zero; *, the difference between means significantly different from zero (*p* < 0.05).

The *J. nigra* chronologies, collected from Montgomery County, ranged in length from 26 to 81 years, with a mean length of 48.8 years. For a two-tailed correlation of annual tree ring widths to climate data, with a sample size of 81 years at a confidence level of 0.05, the critical value for Pearson’s correlation coefficient was 0.216 (36). All climate variables were correlated with the standardized chronology for *J. nigra*, and September’s minimum temperature had the largest negative correlation of −0.262. A linear regression analysis was carried out for September’s minimum temperature as compared with the standardized chronology (**Figure 3A**). The regression analysis showed that approximately 7% (*R* ^2^ = 0.069, df = 81; *p* = 0.018), of the tree’s reduced growth could be attributed to September’s minimum temperature. After subtracting the normalized climate index from the standardized chronology, a Student’s paired *t*-test showed no significant reduction in growth after *L. delicatula* infestation (*t* = 2.056, df = 4; *p* = 0.109). However, the climate-adjusted RWI from 2010 to 2014 was significantly less than from 2005 to 2009 (*t* = 3.559, df = 4; *p* = 0.024; **Figure 3**). The fact that there are differences in the RWI between the two-time intervals in the absence of *L. delicatula* shows that factors other than *L. delicatula* can influence tree regrowth.

#### Liriodendron tulipifera

**Figure 4:**
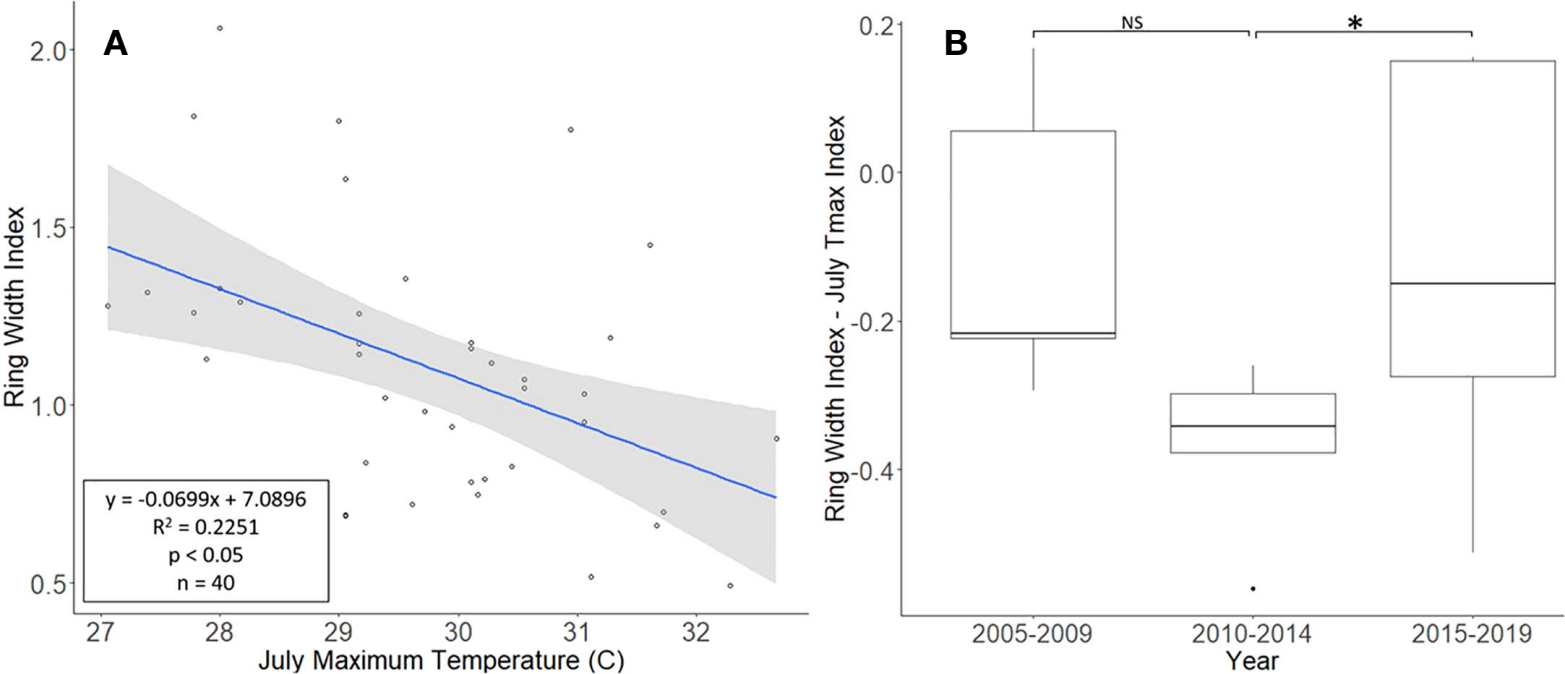
Impact of climate conditions and *Lycorma delicatula* on the ring width index for *Liriodendron tulipifera* in Upper Hanover Township, PA, USA. Regression analysis of *L. tulipifera* ring width index values and July’s maximum temperature. **(A)**; and a comparison of *L tulipifera* ring width index values with the dominant climate variable removed in years before (i.e., 2005 to 2009 and 2010 to 2014) and after (2015 to 2019) the likely start of *L. delicatula* infestation **(B)**. NS, the difference between means not significantly different from zero; *, the difference between means significantly different from zero (*p* < 0.05).

The *L. tulipifera* chronologies, collected from Montgomery County, ranged in length from 17 to 40 years, with a mean length of 25.8 years. For a two-tailed correlation of annual tree ring widths to climate data with a sample size of 40 years at a confidence level of 0.05, the critical value for Pearson’s correlation coefficient was 0.301 (36). All climate variables were correlated with the standardized chronology for *L. tulipifera*, and July’s maximum temperature had the largest negative correlation of −0.474. A linear regression analysis was carried out for July’s maximum temperature as compared with the standardized chronology (**Figure 4A**). The regression analysis showed that approximately 23% (*R* ^2^ = 0.2251, df = 40; *p* < 0.001) of the variation in the RWI could be attributed to July’s maximum temperature. After subtracting the normalized climate index from the standardized chronology, a Student’s paired *t*-test showed a significant reduction in the growth of *L. tulipifera* after *L. delicatula* infestation (*t* = −2.961, df = 4; *p* = 0.042). There was no significant difference in the two uninfested time periods when the dominant climate variable was removed (*t* = 2.288, df = 4; *p* = 0.084; **Figure 4B**).

#### Acer rubrum

The *A. rubrum* chronologies, obtained from Montgomery County, had a high degree of variation in ring width and ranged in length from 19 to 151 years, with a mean length of 61.4 years. None of the seven *Ac. Rubrum* trees sampled correlated well with the master chronology created in COFECHA (**Table 1**) and were excluded from further analysis.

### Impact of chemical treatment on *Ailanthus altissima* growth

The *A. altissima* chronologies, collected from Blue Marsh, ranged in length from 5 to 37 years, with a mean length of 19.4 years. The eight cores did not date well with the master chronology. Discrepancies in the wood could not be identified and the cores were removed from further analysis. All other series dated well in COFECHA, with an interseries correlation of 0.485 (**Table 1**). To remain consistent, we standardized Blue Marsh *A. altissima* chronologies by fitting a horizontal line through the mean, and the distribution of RWIs was found to be approximately normal.

For a two-tailed correlation of annual tree ring widths to climate data, with a sample size of 38 years at a confidence interval of 0.05, the critical value for Pearson’s correlation coefficient was 0.312 (37). All climate variables were correlated against the standardized chronology for *A. altissima* and it was found that June’s and July’s average temperatures had the largest negative correlation, at −0.520 and −0.447, respectively. A linear regression was calculated for June’s and July’s average temperatures as compared with the standardized chronology (**Figure 2C**). The regression analysis showed that approximately 32% (*R* ^2^ = 0.323, df = 38; *p* < 0.001) of reduced tree growth could be attributed to June’s and July’s average temperatures. After subtracting the normalized climate index from the standardized master chronology, the data were broken up into treatments for analysis using a Student’s paired *t*-test (0, 1, and 2 years of insecticide treatment, respectively).

#### No insecticide treatment

**Figure 5:**
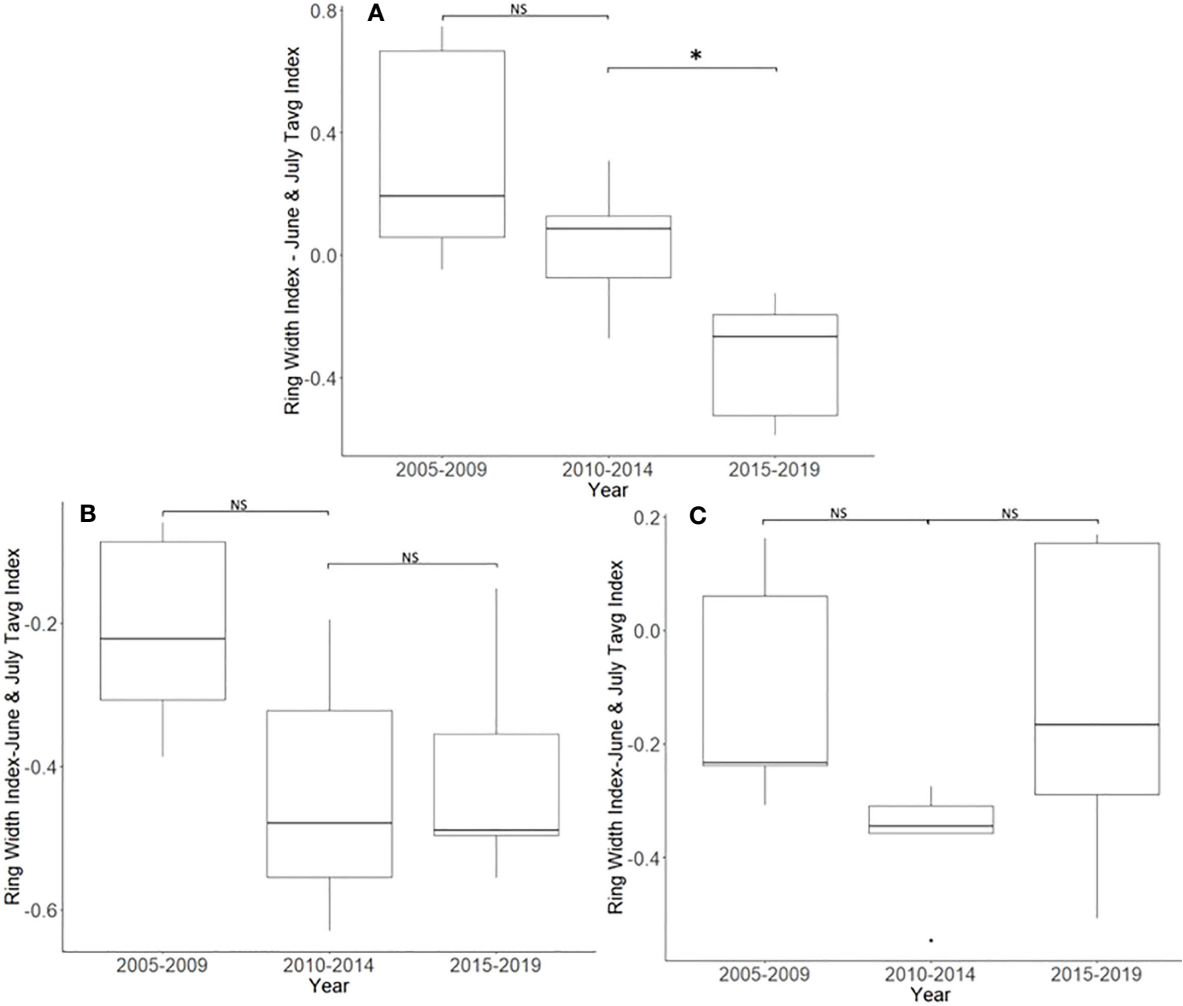
Impact of insecticide treatment and *Lycorma delicatula* on the ring width index with the dominant climate variable removed for *Ailanthus altissima* in Blue Marsh Recreation Area, Berks County, PA, USA. Trees had no insecticide treatment **(A)**, 1 year of treatment **(B)**, or 2 years of treatment **(C)**. Comparisons apply to years before (i.e., 2005 to 2009 and 2010 to 2014) and after (2015 to 2019) the likely start of *L. delicatula* infestations. NS, the difference between means not significantly different from zero; *, the difference between means significantly different from zero (*p* < 0.05).

After accounting for the dominant climate variables, *A. altissima* without insecticide treatment showed a significant reduction in RWI from 2015 to 2019 than from 2010 to 2014 (*t* = 3.513, df = 4; *p* = 0.025). However, no significant difference in climate-adjusted RWIs was found when we compared the two periods presumed to be before the *L. delicatula* invasion period, that is, the period from 2005 to 2009 to that from 2010 to 2014, (*t* = 1.308, df = 4; *p* = 0.261; **Figures 2D**, **5A**).

##### One year of insecticide treatment

After accounting for the dominant climate variables, *A. altissima* that received 1 year of insecticide treatment did not show a significant reduction in climate-adjusted RWI after the presumed introduction of *L. delicatula* (*t* = −0.264, df = 4; *p* = 0.805). Similarly, no significant difference in RWI was found when we compared the two periods before the *L. delicatula* invasion *(t* = 1.818, df = 4; *p* = 0.143; **Figure 5B**).

#### Two years of insecticide treatment

After accounting for the dominant climate variables, *A. altissima* that received 2 years of insecticide treatment did not show a significant reduction in RWI post-*L. delicatula* invasion (*t* = −2.612, df = 4; *p* = 0.059). Similarly, no significant difference in RWI was found when we compared the two preceding periods of uninfested years prior to *L. delicatula* invasion (*t* = 2.153, df = 4; *p* = 0.098; **Figure 5C**).

### Master chronologies

**Figure 6:**
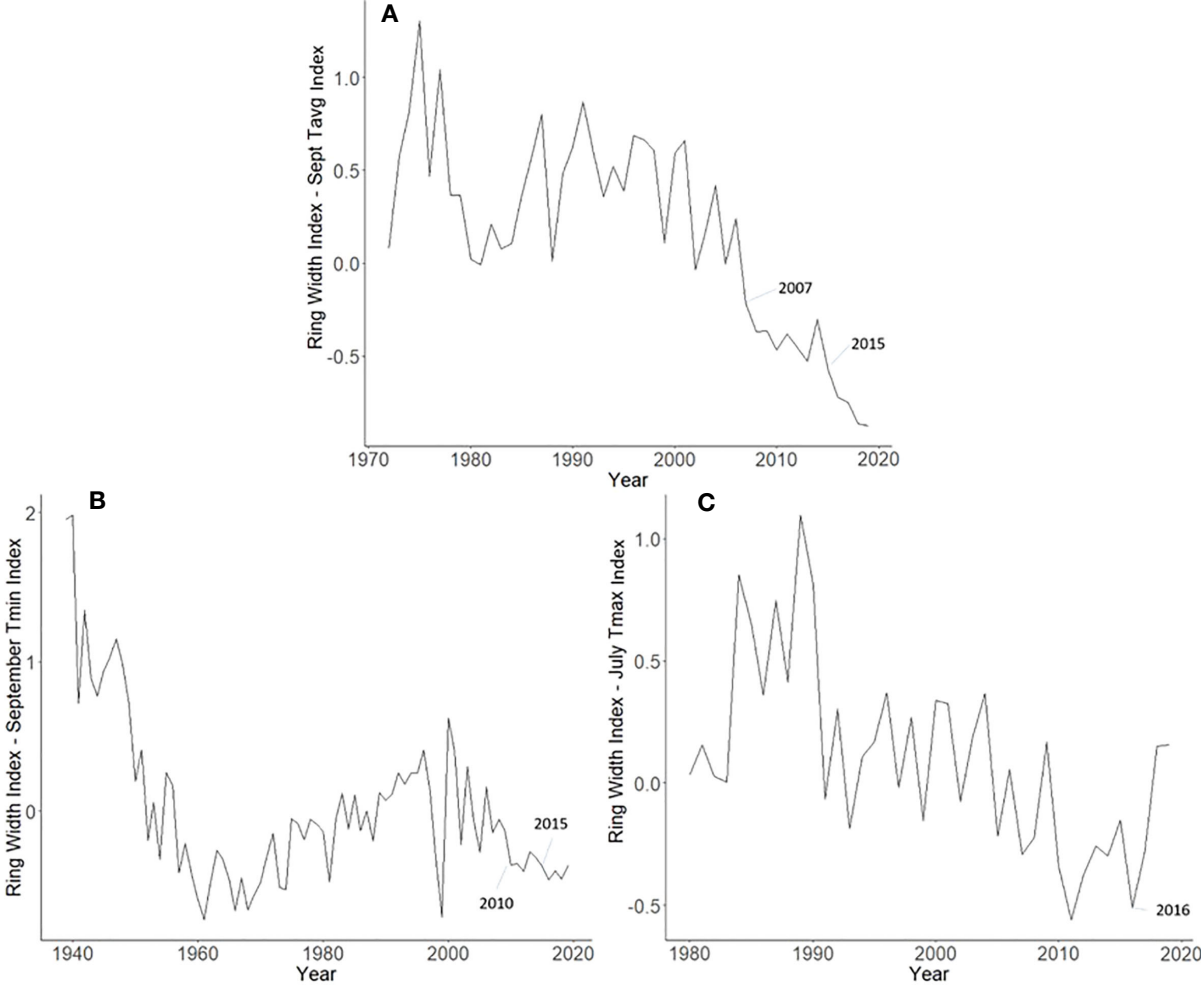
Master chronologies for *Ailanthus altissima* **(A)**, *Juglans nigra* **(B)**, and *Liriodendron tulipifera* **(C)**, showing the annual ring width index with the dominant climate variable removed over the life course of the sampled trees in Upper Hanover Township, PA, USA. *Ailanthus altissima* had suppressed growth in 2007 (likely because of a severe drought that year) and 2015 (potentially from *L. delicatula* feeding). *Juglans nigra* had suppressed growth in 2010, perhaps due to late-season drought. *Liriodendron tulipifera* had a substantial increase in growth rate in 2016, the year *L. delicatula* was confirmed in the area.

Master chronologies indicate differences in growth patterns among the *L. delicatula* hosts examined. *A*. *altissima* had suppressed growth in 2007 (likely from a severe drought that year) and 2015 (potentially from *L. delicatula* feeding) (**Figure 6A**). Interestingly, *J. nigra* had suppressed growth in 2010, perhaps due to a late-season drought, but no negative impacts on growth that could be associated with *L. delicatula* feeding from 2015 onward were found. *L. tulipifera* had a substantial increase in growth rate in 2016, the year *L. delicatula* was confirmed in the area, but no other notable growth observations were made.

## Discussion

### *Lycorma delicatula* impact on *Ailanthus altissima*

We found evidence of *L. delicatula* reducing the annual growth of *A. altissima* at two field sites. Similar impacts on trees have been reported in related systems. Research in Mexico used dendrochronological methods and found that a phloem-feeding scale insect, *Stigmacoccus garmilleri* Foldi (Hemiptera: Stigmacoccidae), negatively affected the growth of oak trees as scale densities increased (37). Similarly, dendrochronological research has shown that *Tsuga canadensis* (L.) (Pinales: Pinaceae) exhibits a sharp reduction in growth immediately following infestation from the xylem feeder *Adelges tsugae* (Annand) (Hemiptera: Adelgidae) (38). Tree ring analysis has also shown that increasing densities of xylem-feeding periodical cicadas, *Magicicada* spp. Davis, can negatively affect the growth of many tree species (14, 31).

Not all observed variations for *A. altissima* RWI seen in the master chronology from the Upper Hanover Site (**Figure 6A**) can be attributed to *L. delicatula* feeding. This result is not surprising because many variables affect tree growth (30). For example, a suppression in *A. altissima* growth occurred prior to *L. delicatula* introduction, beginning in 2007 (**Figure 6A**). This reduction can likely be attributed to a severe drought that occurred during the summer and fall of 2007 in the mid-Atlantic region (39). This drought potentially caused a reduction in growth for the following several years as the trees recovered.

Treating *A. altissima* with the insecticide dinotefuran reduces the impact of *L. delicatula* on tree growth. We were therefore able to compare the radial growth of different *A. altissima* trees over the same time period and location with the only difference being heavy *L. delicatula* feeding influenced by insecticide treatment. In addition, in North America, there are very few arthropod enemies associated with *A. altissima* (40). *Atteva aurea* (Cramer) (Lepidoptera: Attevidae), the *Ailanthus* webworm, has been reported as a non-native herbivore to *A. altissima*, but severe damage has been documented only rarely on seedlings and young saplings (40). All trees sampled in this study measured greater than 25 cm DBH. Therefore, it is unlikely that an additional herbivore of *A. altissima* was responsible for the decreased growth observed in the untreated trees at Blue Marsh. This may indicate, where warranted, that treating high-value trees, such as timber, ornamental, or other economically valuable species, may help to reduce the impacts of *L. delicatula*. In some areas, *A. altissima* is a valuable tree and may benefit from protection. Other tree species, not studied here, may in the future be shown to also be sensitive to feeding by *L. delicatula* (41, 42).

In our experimental design, no trees uninfested with *L. delicatula* were treated with dinotefuran; in theory, the larger tree rings could have been due to the application itself. There have been cases where insecticides have elevated plant functions, including photosynthesis (43). In that study, which featured an evaluation of apple tree response to 33 insecticides, most had no effect on photosynthesis; 12 had an effect, but only two increased photosynthesis. No neonicotinoids were included in that study. However, there has been no evidence reported for elevated plant function by dinotefuran. In fact, this insecticide has been shown to have a negative effect on plant roots (44) and increases oxidative stress in plants (45). It is unlikely, then, that the dinotefuran application itself was responsible for the larger tree ring growth noted in dinotefuran-treated trees.

Our dendrochronological methods did not provide evidence of *L. delicatula* significantly reducing the growth of *J. nigra*. *J. nigra* had sample chronologies that correlated well with their master chronology in COFECHA, indicating that they were accurately dated (**Table 1**). Additional sampling may have discerned a significant difference; a downward trend was apparent. We did detect a significant reduction in the growth of *J. nigra* between the two preceding time periods before we presume *L. delicatula* was introduced. This growth suppression appears to have begun in 2010 (**Figures 3B**, **6B**) and may be the result of a late-season drought affecting the sampling area. A similar decrease in growth can be seen in the master chronology of *A. altissima* from Upper Hanover (**Figure 6A**), but this did not appear to affect significance in the analysis of *A. altissima* cores. The reason for this phenomenon is unclear and beyond the scope of this article; further research is needed.

*Liriodendron tulipifera* also had cores that correlated well with their master chronology in COFECHA (**Table 1**). Once the dominant climate factor of July’s maximum temperature for *L. tulipifera* was removed, there was evidence suggesting a significant increase in growth occurred after *L. delicatula* invasion (**Figure 4B**). This phenomenon could be evidence that some tree species benefit from *L. delicatula* invasion. Yang (2004) tested a hypothesis where he looked at the effect of periodical cicada density on the growth of the American bellflower, *Campanulastru americanum* L. (Asterales: Campanulaceae) (46). He enriched the soil of American bellflowers with different densities of periodical cicada carcasses that resulted in bellflowers in the experimental group having larger seeds and leaves, and higher nitrogen concentrations in leaves than the control group (46).

The impact of *L. delicatula* on *J. nigra* and *L. tulipifera* may still be occurring, despite no impact being detected using our methods. For example, *L. tulipifera* is often less infested than *A. altissima*, and not considered a consistent primary host, whereas *J. nigra* is often seen as a primary host during the fourth instar and early adult life stages of *L. delicatula* (authors’ observation). By contrast, *A. altissima* is frequently documented to host all *L. delicatula* life stages and fed on throughout the entire growing season. Reduced feeding durations on *L. tulipifera* and *J. nigra* may result in growth impacts not being detectable within just 5 years. A larger sample size that includes a diversity of different sites could help clarify if *L. delicatula* does impact growth in non-*A. altissima* tree hosts and ensure that we were not just looking at trees that had escaped herbivory. Furthermore, as *L. delicatula* is often found feeding in the canopies of trees, stem analysis of canopy branches may provide useful information in future studies (40).

Lastly, this difference in impact level between *A. altissima* and *L. tulipifera* and *J. nigra* could be explained by the large number of *A. altissima* at this Upper Hanover Site. *L. delicatula* feeding may have been focused on its preferred host *A. altissima*, to the exclusion of *L. tulipifera* and *J. nigra*, and the results of sampling a site without *A. altissima* may have indicated a significant feeding impact on *J. nigra* and *L. tulipifera*.

## Conclusions

Dendrochronology can be used to identify and quantify long-term *L. delicatula* feeding injury to certain trees, such as *A. altissima*, as it has been used with other phloem feeders or invasive tree-feeding herbivores. We were unable to quantify any negative impact of feeding by *L. delicatula* on *J. nigra* or *L. tulipifera*. Either the radial growth of those species is not affected by *L. delicatula* feeding, or it may be that standard dendrochronology methods may not be the most effective way of identifying a feeding signal and studying the long-term impacts for these tree species. It is possible that the use of quantitative wood anatomy and the hydrologic conductance measured by pore size could be used as a better measure of insect injury. It is also possible that these tree species are simply not as affected by *L. delicatula* feeding. Basal insecticide applications of dinotefuran appear to reduce and prevent damage to *A. altissima* trees that experience heavy feeding by *L. delicatula.*

## Data availability statement

The raw data supporting the conclusions of this article will be made available by the authors, without undue reservation.

## Ethics statement

The manuscript presents research on animals that do not require ethical approval for their study.

## Author contributions

AD performed, analyzed, and wrote the original version. DP and TK supervised research and advised student development. SS and TL participated in the design and analysis. KM assisted in analysis and writing. BW assisted in the field research carried out in Pennsylvania. JS assisted in core analysis. All authors contributed to the article and approved the submitted version.
